# Mifepristone's Efficacy for Symptomatic Relief and Size Reduction in Uterine Fibroids: A 2023 Prospective Observational Study at Rabia Balkhi Hospital, Afghanistan

**DOI:** 10.7759/cureus.73432

**Published:** 2024-11-11

**Authors:** Bibi Sarah Yousofzai, Malalai Alami, Sreeja Krishna Sheela, Muhammad Subhan, Ruqiya Bibi, Ashik Ali, Mayankkumar D Dhakecha, Tooba Zafar, Lavinya Vasudevan, Muaz Shafique Ur Rehman

**Affiliations:** 1 Obstetrics and Gynaecology, Cure Hospital, Be Team International, Kabul, AFG; 2 Obstetrics and Gynaecology, Rabia Balkhi Hospital (RBH) National Hospital, Kabul, AFG; 3 Medicine and Surgery, Mandya Institute of Medical Sciences, Mandya, IND; 4 Medicine, Jinnah Hospital Lahore, Allama Iqbal Medical College, Lahore, PAK; 5 Medicine, Allama Iqbal Medical College Lahore, Pakistan, Lahore, PAK; 6 Paediatrics and Child Health, SRM Medical College, Chennai, IND; 7 Medicine, Government Medical College & Hospital, Surat, IND; 8 Internal Medicine, Sheikh Zayed Medical College, Rahim Yar Khan, PAK; 9 Public Health, Monash University, Subang Jaya, MYS; 10 Internal Medicine, Jinnah Hospital Lahore, Lahore, PAK

**Keywords:** antiprogestogen, fibroids, leiomyomas, menorrhagia, mifepristone

## Abstract

Background

Uterine fibroids (leiomyomas) are common benign tumors, affecting 70-80% of women by age 50, and can cause symptoms such as heavy bleeding, pelvic pain, and pressure, significantly impacting the quality of life. In severe cases, fibroids may lead to infertility or miscarriage, making their management a key healthcare challenge. Traditional treatments, including hysterectomy, may not be suitable for women wishing to preserve fertility or avoid surgery. Mifepristone, an antiprogestogen, has shown promise as a non-invasive alternative by blocking progesterone, which promotes fibroid growth. It has been proven to reduce fibroid size and alleviate symptoms, offering a valuable option for women seeking alternatives to invasive procedures.

Objective

This study aimed to evaluate the efficacy of mifepristone in providing symptomatic relief and reducing fibroid size in patients with uterine fibroids at Rabia Balkhi Hospital, Kabul, Afghanistan, in 2023.

Methodology

A descriptive, prospective study was conducted from January to June 2023, enrolling 20 women with symptomatic uterine fibroids. The inclusion criteria were women aged 30-55 years with clinically diagnosed fibroids causing symptoms such as heavy bleeding or pelvic pain. Exclusion criteria included pregnancy, active infections, or other uterine pathologies. Patients were treated with 25 mg of mifepristone daily for three months. Follow-up assessments were conducted at one, two, and three months to monitor clinical outcomes, including changes in bleeding, fibroid size (measured by ultrasound), and hemoglobin levels.

Results

Twenty patients (6.25%) with symptomatic fibroids were treated with mifepristone. The majority of cases were in patients aged 46-55 years (50%). Treatment led to a reduction in bleeding in 78% of patients, a decrease in fibroid size in 42.6%, and a reduction in menorrhagia in 44%, evidenced by fewer daily sanitary products used. Hemoglobin levels improved in 33.5% of patients, with an average post-treatment level of 11.8 ± 1.4 g/dL. Statistical significance was set at p < 0.05, supporting mifepristone’s efficacy in managing symptomatic fibroids and enhancing patient quality of life.

Conclusion

Our study concludes that mifepristone at 25 mg is an effective and accessible treatment for fibroids, the same as other published evidence. It offers a practical and economical alternative to surgery, reducing bleeding, inhibiting fibroid growth, and decreasing fibroid size. These findings have significant public health implications, reassuring patients and healthcare providers about the accessibility of effective fibroid treatment.

## Introduction

Uterine fibroids, or leiomyomas, are a significant health issue as they are the most common benign tumors of the uterus, originating from smooth muscle cells [1,2]. They affect 20-30% of women of reproductive age and up to 80% of women aged 30-50 years, with a higher prevalence in Black women [2]. Risk factors include genetic predisposition, reproductive age, hormonal influences, heredity, and race [3]. Fibroids, which lack a capsule, can vary in size and location within the uterus and are classified as submucosal, intramural, subserosal, or cervical [4]. Genetic factors significantly influence their development, making certain populations, such as Black women and those of Syrian descent, more susceptible [3,4]. The symptoms, which depend on the size and location of the fibroids, can have a profound impact on women's health, including heavy menstrual bleeding, abnormal vaginal bleeding, anemia, constipation, abdominal pain, pelvic pain, dyspareunia, and pressure-related symptoms [4,5]. Diagnosis typically involves patient history, symptoms, and examinations such as ultrasound, MRI, and hysteroscopy [5]. Management options include medical treatments, such as mifepristone, and surgical interventions including hysterectomy, aiming to reduce symptoms, correct anemia, and decrease fibroid size [5,6]. Mifepristone is an antiprogestogen that works by blocking the action of progesterone, a hormone that plays a key role in fibroid growth [6,7]. Progesterone promotes the proliferation of smooth muscle cells within the fibroid, and by inhibiting this action, mifepristone reduces fibroid size and alleviates symptoms such as heavy bleeding [7,8]. Given the significant impact of uterine fibroids on women's health, there is a need for non-invasive alternatives to surgery [8,9]. Mifepristone offers a potential solution by reducing fibroid size and symptoms with fewer risks and a shorter recovery time [9,10]. This study aims to determine the effectiveness of mifepristone in treating uterine fibroids at Rabia Balkhi Hospital, Kabul, Afghanistan, in 2023.

## Materials and methods

This study aimed to evaluate the efficacy of mifepristone as a treatment for symptomatic uterine fibroids in women presenting at Rabia Balkhi Hospital in 2023. Uterine fibroids, defined here as benign, non-cancerous growths originating from the smooth muscle tissue of the uterus (myometrium), can lead to significant symptoms, including abnormal bleeding and pelvic discomfort. Mifepristone, an anti-progestational agent, was utilized in this study at a daily dose of 25 mg to assess its impact on reducing fibroid size and controlling uterine bleeding. Transvaginal ultrasound (TVUS), a widely utilized and reliable imaging technique for gynecological assessments, was used to accurately measure fibroid dimensions both at baseline and throughout treatment.

Study population

The study population consisted of women presenting with gynecological issues who were subsequently diagnosed with uterine fibroids. From this group, a sample of 20 patients with symptoms directly attributable to fibroids was enrolled. Participants’ ages ranged from 25 to 55 years, with a mean age of 43.7 ± 7.2 years. Age distribution was as follows: 50% were between 46 and 55 years (mean age: 50.3 ± 3.1 years), 30% between 36 and 45 years (mean age: 40.5 ± 2.4 years), and 20% between 25 and 35 years (mean age: 30.7 ± 2.1 years). This demographic distribution provided a broad representation of the typical age range affected by symptomatic fibroids.

Inclusion and exclusion criteria

Patients had to meet specific inclusion criteria for this study. Eligible participants were women aged 25-55 with symptomatic uterine fibroids, hemoglobin levels above 8 g/dL, and a willingness to undergo mifepristone treatment. Strict exclusion criteria were also applied to ensure safety and treatment focus. Women were excluded if they were pregnant or seeking pregnancy, had known or suspected malignancies (e.g., uterine or ovarian cancer), severe anemia (hemoglobin < 8 g/dL), other uterine pathologies (e.g., adenomyosis or endometrial hyperplasia), or a history of hypersensitivity to mifepristone.

Informed consent

Informed consent was obtained from all participants before their involvement in the study. The participants were provided with detailed information about the study’s purpose, procedures, potential benefits, and associated risks. Written consent was obtained after patients had the opportunity to ask questions and were fully informed. Participants were assured that their confidentiality would be protected, and they were informed of their right to withdraw from the study at any point without consequence to their ongoing care.

Data collection

Data collection involved comprehensive methods to ensure an accurate assessment of the effects. Detailed patient histories were recorded, focusing on baseline symptoms, medical history, and prior gynecological treatments. Clinical assessments included systemic and gynecological examinations to establish baseline measurements for fibroid size, blood loss, and hemoglobin levels. These assessments were conducted during initial evaluations and follow-up visits to monitor changes over time. Fibroid size was measured using ultrasound imaging, and to assess menstrual blood loss, patients were asked to estimate the number of sanitary products used per day during menstruation, categorizing them as lightly, moderately, or fully soaked. This simple method allowed for a consistent and practical evaluation of menstrual blood loss throughout the study, conducted at the initial visit and during follow-up appointments to track treatment-related changes. Hemoglobin levels were measured through standard blood tests to assess changes in anemia related to fibroid-induced blood loss.

Data analysis

The effectiveness of mifepristone was assessed by comparing baseline and follow-up data on fibroid size, bleeding volume, and hemoglobin levels. Fibroid volume changes were measured via ultrasound, while reduced menstrual blood loss was evaluated by tracking the number of sanitary products used per day. Hemoglobin levels were monitored to assess improvements in anemia related to reduced bleeding. This prospective study applied descriptive statistics, including mean, median, standard deviation, and range, to provide a comprehensive analysis of mifepristone’s impact on symptom relief and quality of life for patients with uterine fibroids.

## Results

To determine the required sample size, a significance level of 0.05 and a power of 80% were set, with an anticipated effect size of 0.5, based on prior studies suggesting moderate treatment impact. This calculation aimed to ensure an adequate sample representation of symptomatic fibroid cases, balanced against hospital resource constraints and ethical considerations. In 2023, a prospective study at Rabia Balkhi Hospital evaluated the efficacy of mifepristone in 20 patients with symptomatic uterine fibroids, representing 6.25% of 320 diagnosed fibroid cases, among a total of 25,122 gynecological patients (Table 1). The majority of study participants were in the 46-55 age group (50%), followed by 36-45 (30%) and 25-35 (20%) age groups. Mifepristone demonstrated a significant therapeutic effect: 78% (n=16) of participants experienced reduced bleeding, 44% (n=9) reported reduced menorrhagia, 42.6% (n=9) had a reduction in fibroid size, and 33.5% (n=7) showed an increase in hemoglobin levels. The most significant size reduction was observed in fibroids measuring 3-7 cm (69.7%; n=14), with smaller fibroids (<3 cm) showing 10% reduction (n=2) and larger fibroids (>7 cm) showing 18.3% reduction (n=4). Fibroid location was predominantly subserosal (65.5%; n=13), followed by submucosal (15.9%; n=3) and intramural (8.6%; n=2). Minor side effects were experienced by 72% of participants (n=14), including temporary amenorrhea, which resolved post-treatment.

**Table 1:** Summary of Mifepristone Study Results in Uterine Fibroids at Rabia Balkhi Hospital (2023)

Category	Subcategory	Mean ± SD/Median (Range)	Percentage (%)	N (Value)
Total No. of Patients	Gynecological Patients	N/A	100%	25,122
Diagnosed Fibroid Cases		N/A	1.27%	320
Study Participants		N/A	6.25%	20
Age Distribution	46-55 years	50.3 ± 3.1 years	50%	10
	36-45 years	40.5 ± 2.4 years	30%	6
	25-35 years	30.7 ± 2.1 years	20%	4
Effectiveness of Mifepristone	Reduction in Bleeding	-	78%	16
	Reduction in Fibroid Size	-	42.6%	9
	Reduction in Menorrhagia	-	44%	9
	Increase in Hemoglobin	11.8 ± 1.4 g/dL (post-treatment)	33.5%	7
Fibroid Size Reduction	Less than 3 cm	-	10%	2
	3-7 cm	-	69.7%	14
	Larger than 7 cm	-	18.3%	4
Fibroid Location	Subserosal	-	65.5%	13
	Intramural	-	8.6%	2
	Submucosal	-	15.9%	3
Side Effects	Minor Side Effects	-	72%	14
	Amenorrhea (Resolved)	-	72%	14

## Discussion

Uterine fibroids, also referred to as leiomyomas, are benign tumors found on the smooth muscle layer of the uterus that affect many women during their reproductive years [1,2]. Fibroids can have significant clinical relevance due to their potential to cause symptoms that include heavy menstrual bleeding (menorrhagia), pelvic pain, and pressure symptoms, as well as reproductive concerns, such as infertility or repeated pregnancy loss [2,3]. Fibroid management encompasses various approaches, from conservative approaches for those experiencing minimal symptoms to more aggressive medical and surgical treatments [4,5]. Medical management typically seeks to relieve symptoms and shrink fibroid size [5]. At the same time, surgical interventions such as myomectomy or hysterectomy should only be utilized for more serious cases or when medical therapy fails [6]. Unfortunately, surgery involves risks that include adhesion formation, infertility, and decreased quality of life [7].

Mifepristone (RU 486), a synthetic steroid, is a promising medical therapy due to its unique antiprogestogen and anti-glucocorticoid effects [1-4]. Unlike other treatments, mifepristone inhibits progesterone secretion while blocking progesterone receptors, key factors in maintaining fibroids' growth and maintenance [7,8]. It also increases oxytocin and prostaglandin receptor sensitivity, promoting uterine contractions [9]. Importantly, it does all this without permanently changing endometrium cells' structure, making mifepristone an SPRM that leaves no permanent changes behind [8-10]. In the context of fibroid management, mifepristone can be used as a noninvasive alternative to surgical interventions, particularly for patients who are not suitable candidates for surgery or wish to avoid its associated risks [9,10].

Numerous clinical studies have evaluated the efficacy of mifepristone for treating fibroids [8-14]. One randomized clinical trial in Havana, Cuba, with 220 patients aged 45-55, revealed a 43% reduction in fibroid volume; an equal decrease was noted in uterine volume reduction and a 79% decrease in blood loss [8]. Hemoglobin levels increased by 34%, and 66% of patients experienced amenorrhea during treatment [8]. A prospective clinical study in 2022 at a public hospital in New Delhi, India, involved enrolling 70 women aged 25-55 who received three monthly doses of 25 mg oral mifepristone; most (62.77%) fell within the 41-51 age range [9]. The age distribution of participants was as follows: 5.7% were within 21-30 years, 19.8% were aged 31-40 years, 62.77% fell within the 41-50 years range, and 4.2% were within 52-60 years [9]. Of all participants, 69% were married, while 31% were unmarried [9]. Submucosal fibroids were present in 16.7% of cases, while intramural fibroids comprised 73% [9]. They presented with symptoms such as menorrhagia, dysmenorrhea, and dyspareunia; of all patients examined, 14.2% had fibroids smaller than 3 cm, while 37.26% had fibroids between 3 and 7 cm in size - 67.16% had larger than 7 cm fibroids [9]. Overall, participants reported a 35% decrease in fibroid size, a 26.4% reduction in uterine size, and an 86.7% decline in dysmenorrhea [9]. Furthermore, an increase in hemoglobin (34.3%) and a decrease in bleeding (84%) were noted; 78.57% reported no side effects after using this intervention [9].

Malik et al. conducted a descriptive, retrospective study between 2016 and 2019 at Liaqat Memorial Hospital, Kohat, Pakistan [10]. The study evaluated the effectiveness of mifepristone in treating uterine fibroids [10]. A total of 160 patients participated, with 79.5% aged between 43 and 53 years [10]. The main complaint among participants was metrorrhagia (87.51%), followed by menorrhagia (32.22%) [10]. The study found a 41.3% decrease in fibroid volume, a 75% reduction in blood loss, a 32% increase in hemoglobin levels, and an 86.7% reduction in dysmenorrhea symptoms [10]. An expert consensus by Kriplani et al. underscores the safety of low-dose (25 mg) mifepristone for managing fibroids [11]. According to this consensus statement, low-dose mifepristone can be used both as a pre-operative adjunct treatment to shrink fibroids before surgery and as a post-op treatment to prevent future recurrences [11]. The consensus further assures that prolonged usage of mifepristone is safe, with an extremely low cumulative incidence rate post-surgery, providing a strong sense of reassurance and confidence in its use [11]. Desai et al. evaluated mifepristone's effectiveness and safety in decreasing uterine fibroids' size and symptoms [12]. This prospective study enrolled 20 premenopausal women aged 18 years or older from Nanavati Max Super Speciality Hospital in Mumbai, India [12]. Participants averaged 39.75 years old with an average BMI of 27.58 kg/m^2^ [12]. The research team discovered that mifepristone significantly reduced uterine volume by 75%, increasing hemoglobin levels from 9 gm/dL initially to 12.51 gm/dL at six months [12]. Pain intensity, measured with a visual analog scale (VAS) score, steadily declined between baseline and three months - all 20 patients reported a complete absence of pelvic pain by six months [12].

These additional studies bolster mifepristone's efficacy in managing uterine fibroids and highlight its potential as a noninvasive treatment option [8-15]. Comparing our study to those cited, several similarities and distinctions become evident. Rabia Balkhi Hospital reported a 42.6% reduction in fibroid size, with a p value <0.05, similar to what was observed in Cuba and Pakistan (43 and 41.3, respectively). Our study's reduction of bleeding (78%) closely aligns with findings from Cuba (79%) and India (84%). Hemoglobin levels rose 33.5% - comparable to increases reported by Cuba (34.3%) and India (34.5%).

Limitations 

Our study’s small sample size, short follow-up period, and lack of participant diversity limit its generalizability and assessment of long-term outcomes. The reliance on self-reported symptoms and the absence of a control group introduce potential bias, impacting our ability to isolate mifepristone’s effects. Future studies should consider larger, multicenter designs with diverse populations, control groups, and extended follow-up for a more comprehensive evaluation of mifepristone's long-term efficacy and safety.

## Conclusions

Our study at Rabia Balkhi Hospital in 2023 demonstrated that 25 mg of mifepristone is an effective and accessible treatment for uterine fibroids. The results showed significant reductions in fibroid size, bleeding, menorrhagia, and a notable increase in hemoglobin levels. These findings suggest that mifepristone can be a practical and economical alternative to surgical interventions, providing substantial relief from symptoms and improving overall patient health. The study's outcomes have important public health implications, reassuring patients and healthcare providers about the efficacy and safety of mifepristone in managing uterine fibroids. Further research with larger sample sizes and extended follow-up periods is recommended to validate these findings and explore the long-term benefits and safety of mifepristone in diverse populations.
